# Puromycin aminonucleoside‐induced podocyte injury is ameliorated by the Smad3 inhibitor SIS3

**DOI:** 10.1002/2211-5463.12916

**Published:** 2020-07-07

**Authors:** Lina Jiang, Hong Cui, Jie Ding, Aijun Yang, Yingchao Zhang

**Affiliations:** ^1^ Pediatric Department Beijing Friendship Hospital Capital University of Medical Sciences Beijing China; ^2^ Pediatric Department Peking University First Hospital Beijing China

**Keywords:** podocyte injury, puromycin aminonucleoside, smad3, transgelin

## Abstract

Smad3 signaling and transgelin expression are often activated during puromycin aminonucleoside (PAN)‐induced podocyte injury. Here, we investigated whether the Smad3 inhibitor SIS3 can ameliorate damage to injured podocytes. A model of PAN‐induced podocyte injury was constructed using the MPC5 cell line. The effects of SIS3 on the expression of the podocyte cytoskeletal proteins transgelin, p15*^INK4B^*, phosphor‐smad3, phosphor‐JAK/stat3, the apoptotic marker cleaved caspase 3, and c‐myc were investigated using western blot. The distribution of F‐actin in PAN‐induced podocyte injury was observed under an immunofluorescence microscope. PAN‐induced podocyte injury altered the distribution of F‐actin and transgelin, and colocalization of these two proteins was observed. Transgelin expression and Smad3 phosphorylation were increased in the MPC5 cell line with prolonged PAN treatment. In addition, c‐myc expression, p15*^INK4B^*, and JAK phosphorylation were all increased after treatment with PAN. Treatment with the Smad3 inhibitor SIS3 reversed these phenomena and protected against PAN‐induced podocyte injury. Moreover, stimulating podocytes directly with TGFβ‐1 also led to enhanced expression of transgelin or phosphor‐JAK/stat3, and this could be inhibited by SIS3. In conclusion, transgelin expression was induced through the Smad3 signaling pathway during PAN‐induced podocyte injury, and the resulting abnormal distribution of F‐actin and the enhanced expression of transgelin could be reversed by blockade of this pathway.

## Graphical abstract

Smad3 signaling and transgelin expression are often activated during puromycin aminonucleoside (PAN)‐induced podocyte injury. Here, we investigated whether the Smad3 inhibitor SIS3 can ameliorate damage to injured podocytes. Transgelin expression was induced through the Smad3 signaling pathway during PAN‐induced podocyte injury, and the resulting abnormal distribution of F‐actin and enhanced expression of transgelin was reversed by blockade of this pathway.

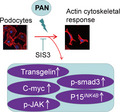

**Abbreviations**
FPs: foot processes
FSGS: focal segmental glomerulosclerosis
MCNS: minimal change nephrotic syndrome
MN: membranous nephropathy
PAN: puromycin aminonucleoside
TGF‐β1: transforming growth factor β1

Podocytes, also known as terminally differentiated glomerular visceral epithelial cells, have giant cell bodies and outstretched foot processes (FPs), which are required to maintain the integrity of the slit diaphragm and are important parts of the glomerular filtration barrier [1, 2]. FPs have been reported to exhibit dynamic and reversible changes during some kidney diseases with abnormal albuminuria, such as human minimal change nephrotic syndrome (MCNS) [3, 4, 5]. Numerous studies have shown that the severity of proteinuria is related positively to FP width and the degree of FP effacement and fusion [6, 7]. The extent of FP fusion in patients with nephrotic syndromes decreases after treatment, referred to as FP retraction during podocyte remodeling. Research on this remodeling process will shed light on the mechanism underlying the occurrence of proteinuria and its molecular target. However, the mechanism of FP retraction remains unclear. Cytoskeletal proteins, such as actin filament (F‐actin) and actin‐binding protein, play important roles in maintaining the integrity of normal podocyte morphology and the full biological function of these cells [8, 9]. Currently, however, whether some cytoskeletal molecules are critical for the occurrence, development, and resolution of proteinuria, and for podocyte remodeling, remains unclear [10]. Investigation of the molecular regulatory mechanism of cytoskeletal actin remodeling will aid understanding of the pathogenesis of proteinuria and provide new targets for its treatment.

Puromycin aminonucleoside (PAN) induces podocyte injury *in vitro* and *in vivo* [11], and this damage is often accompanied by F‐actin disruption and glomerular dysfunction. The podocyte cytoskeletal proteins, which are important for the maintenance of FP morphology, are critically regulated during podocyte injury and proteinuria [12, 13]. Many signal transduction pathways, such as those of mitogen‐activated protein kinase [10, 14], extracellular signal‐regulated kinase [15], phosphatidylinositol 3‐kinase [16], and nuclear factors of activated T cells [17], have been demonstrated to be involved in PAN‐induced podocyte injury. In a rat model of PAN‐induced kidney injury, transforming growth factor β1 (TGF‐β1) and phospho‐Smad2/3 expression was upregulated [18]. p15*^INK4B^* was reported to be the downstream protein in the TGF‐β1 signaling pathway [19]. PAN‐induced podocyte injury could be alleviated by the modulation of TGF‐β1 signaling, but whether Smad3 is involved in PAN‐induced podocyte cytoskeletal disorder remains unknown.

In a previous study [20], we identified nine differentially expressed cytoskeletal genes in a rat model of PAN‐induced kidney disease using genome‐wide DNA microarray analysis. The differential expression of *transgelin*, *survivin*, *arp2*, *cytokeratin 7*, and *vinculin* was confirmed by real‐time RT/PCR and western blotting. Similar expression and distribution changes were detected in patients with proteinuric renal diseases and in PAN‐treated podocytes. The expression of the newly identified 22‐kDa cytoskeletal actin‐binding protein transgelin was upregulated most obviously among the five identified molecules. Transgelin is expressed and distributed abnormally in kidney tissue in the contexts of MCNS, focal segmental glomerulosclerosis (FSGS), and membranous nephropathy (MN) [21, 22]. This protein is also localized in kidney podocytes of rats and children with kidney disease [23]. How it participates in podocyte injury and the development of proteinuria, however, remains unclear.

The current study was conducted to investigate the molecular mechanism of PAN‐induced podocyte injury, to further our understanding of the pathogenesis of proteinuria and to provide novel targets for therapeutic intervention. We hypothesized that the newly identified transgelin protein was a key podocyte molecule important for FP retraction. The expression and distribution of cytoskeletal proteins, such as F‐actin and transgelin, were regulated by the STAT3 pathway.

## Materials and methods

### Cell culture and drug treatment

Conditionally immortalized mouse podocyte lines (MPC5; gifts from P. Mundel, Harvard University) were cultured as described previously [24]. In brief, podocytes were grown in Roswell Park Memorial Institute (RPMI) 1640 medium (Gibco, Grand Island, NY, USA) containing 10% FCS (Gibco) and 10 U·mL^−1^ γ‐interferon (PEPRO Tech, Rocky Hill, NJ, USA) at 33 °C. To induce differentiation, podocytes were cultured at 37 °C with 0.5% FCS, but no γ‐interferon, for about 1 week. Then, podocytes were seeded into 60‐mm Petri dishes containing 10 μg·mL^−1^ type I collagen (Sigma, St. Louis, MO, USA) and maintained in RPMI 1640 medium at 37 °C.

Podocyte injury was induced by PAN as described elsewhere [25]. Briefly, after the differentiated podocytes had grown to about 60% confluence, the culture medium was changed to serum‐free RPMI 1640 containing 0.5% serum replacement for synchronization, and the podocytes were treated immediately with PAN (50 μg·mL^−1^; Sigma). The total cell lysates were then extracted for the detection of transgelin protein expression by western blotting. SIS3 (5 μm; Calbiochem, San Diego, CA, USA), a Smad3 inhibitor, was added to the culture medium 30 min before stimulation with PAN.

### Immunofluorescence

The differentiated podocytes were grown on glass slides coated with type I collagen in serum‐free RPMI 1640 medium containing 0.5% serum for 6 h and then treated with PAN (50 μg·mL^−1^) for 24, 48, and 72 h. The following experimental groups were defined: control, PAN treatment, SIS3 treatment, and combined PAN and SIS3 treatment. For the group treated with the Smad3 inhibitor SIS3, 80% confluent differentiated podocytes were first cultured with 0.5% serum for 6 h and then treated with 5 μm SIS3 for 30 min and with 50 μg·mL^−1^ PAN for 48 h to induce podocyte injury.

Cells were fixed with 4% paraformaldehyde for 15 min at room temperature and then washed three times with PBS for 5 min each. The fixed podocytes were incubated with 0.5% Triton X‐100 for 15 min and then washed three times with PBS for 5 min each. After blocking with 5% BSA for 30 min at room temperature, the cells were incubated with Hoechst (1 : 600, final concentration 0.2 μg·mL^−1^; Santa Cruz Biotechnology, Santa Cruz, CA, USA) and wheat germ agglutinin antibody (1 : 500, final concentration 5 μg·mL^−1^; Abcam, Cambridgeshire, UK) for 1 h at room temperature, away from light. Then, the cells were washed three times with PBS for 5 min each and incubated with F‐actin (Invitrogen, Carlsbad, CA, USA) or transgelin antibody (Santa Cruz Biotechnology) overnight at 4 °C. After washing, the cells were incubated with secondary antibodies (Invitrogen). Then, to prevent fluorescence quenching, the cells were washed with distilled water to remove salts from the PBS. After being mounted with 15% Mowiol, the cells were observed under a laser scanning confocal microscope (Fluoview FV1000; Olympus, Tokyo, Japan). The imagequant software (Olympus) was used for image analysis and processing.

### Western blotting

Podocytes were treated with PAN (50 μg·mL^−1^; Sigma) for 12 h for the detection of transgelin protein expression by western blotting. For the detection of Smad3 phosphorylation, podocytes were treated with the same PAN concentration for 2, 5, 10, 15, and 30 min. For some experiments, the Smad3 inhibitor SIS3 was added to the culture medium 30 min before stimulation with PAN.

Total cell lysates were harvested in ice‐cold lysis buffer (P001; Ukzybiotech Company, Beijing, China) containing an inhibitor cocktail (04693116001; Roche, Basel, Switzerland). Proteins were loaded equally (60 μg) and separated by 12% SDS/PAGE. The gels were transferred onto poly(vinylidene difluoride) membrane (Millipore, Billerica, MA, USA) by wet blotting. The membrane was blocked with 5% fat‐free milk for 1 h, followed by incubation with primary antibodies against Smad3 (1 : 750, 9523T; Cell Signaling Technology, Beverly, MA, USA), phosphor‐Smad3 (1 : 1000, 9520T; Cell Signaling Technology), transgelin (1 : 1000, Ab155272; Abcam), p15*^INK4B^* (1 : 1000, Ab53034; Abcam), phosphor–Janus kinase (JAK; 1: 1000, 3331S; Cell Signaling Technology), phosphor‐STAT3 (1 : 1000, 9145T; Cell Signaling Technology), caspase 3 (1 : 1000, 9661T; Cell Signaling Technology), and c‐myc (1 : 1000, 9402S; Cell Signaling Technology). After incubation with horseradish peroxidase‐conjugated secondary antibodies (1 : 4000, 115‐035‐003 and 111‐035‐003; Jackson ImmunoResearch, Lancaster, PA, USA), signals were visualized with Super Signal West Pico enhanced chemiluminescent substrate (WBKLS0500; Millipore) and analyzed with the AlphaImager gel imaging analysis system (TM2200; Shimadzu, Kyoto, Japan). The house‐keeping protein β‐actin (1 : 1000, TA‐09; ZsBio Company, Beijing, China) was used as an internal control.

### Statistical analyses

The spss 22.0 software (IBM, Armonk, NY, USA) was used to analyze western blotting bands with gray values. The data are presented as means ± SE from at least three independent experiments. To compare three or more groups, we used one‐way analysis of variance followed by multiple comparison tests (least significant difference test in cases of equal variance, Dunnett's T3 test in cases of unequal variance). For comparison of two groups, the independent‐sample *t*‐test was used. Statistical analysis was performed at a significance level of *P* < 0.05.

## Results

### Abnormal distribution of transgelin and F‐actin was observed in PAN‐induced podocyte injury

**Fig. 1:**
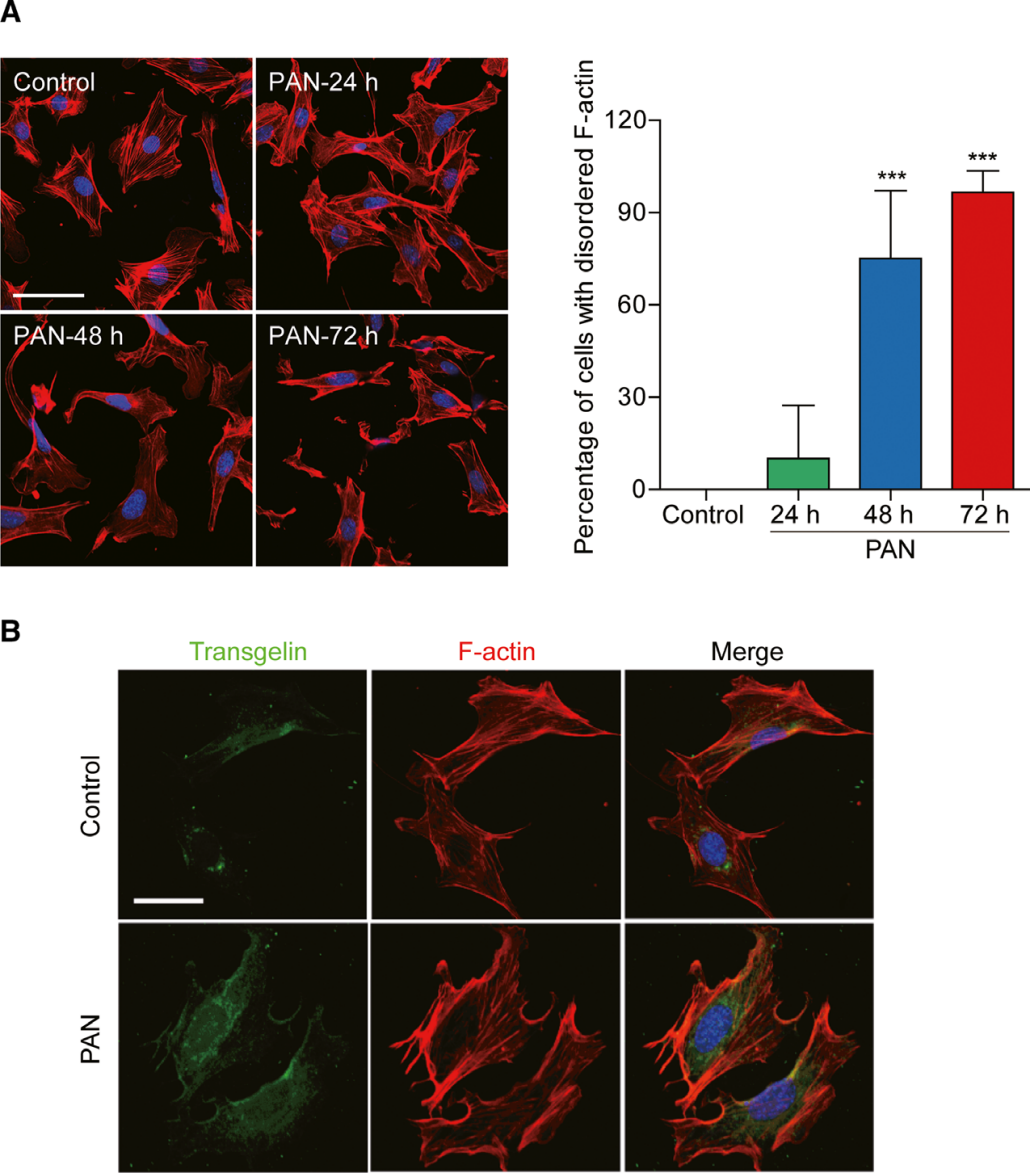
Distribution of podocyte cytoskeletal proteins after PAN treatment. Differentiated MPC5 podocytes were grown on glass slides coated with type I collagen in serum‐free RPMI 1640 medium containing 0.5% serum for 6 h and then treated with PAN (50 μg·mL^−1^) for 24, 48, and 72 h. Cells were incubated with Hoechst for nuclear staining (blue signal). Then, the cells were incubated with F‐actin antibody (red signal, A) or co‐incubated with F‐actin and transgelin antibodies (green and red signals, B). The percentage ratios of disordered cell numbers to total cell numbers in the four groups are summarized in a histogram (*n* = 6, means ± SEM). ***P < 0.001 vs. control. Disordered arrangement of the cytoskeleton and colocalization of F‐actin and transgelin was observed after treatment with PAN. Scale bar = 50 μm.

In normal control MPC5 cells, the red signal was distributed evenly in cytoplasm and radially in podocyte FPs, formed wispy clusters of filaments, indicating that F‐actin was distributed in the integrated cytoskeleton (Fig. 1A). PAN treatment (for 24, 48, and 72 h) resulted in podocyte cytoskeleton damage, FP retraction, and pathological remodeling; the cytoskeletal network disappeared and cell integrity was damaged.

In normal MPC5 cells, transgelin protein was expressed weakly and distributed evenly in the cytoplasm (Fig. 1B). After treatment with PAN for 48 h, the intensity of green fluorescence was enhanced obviously, indicating that transgelin expression was induced. The expression of transgelin after PAN treatment was located mainly in the cell membranes. PAN treatment also disordered the arrangement of the podocyte microfilament cytoskeletal protein F‐actin, characterized by depolymerization and disappearance; transgelin and F‐actin were colocalized.

### Transgelin expression and Smad3 phosphorylation were increased in PAN‐induced podocyte injury

**Fig. 2:**
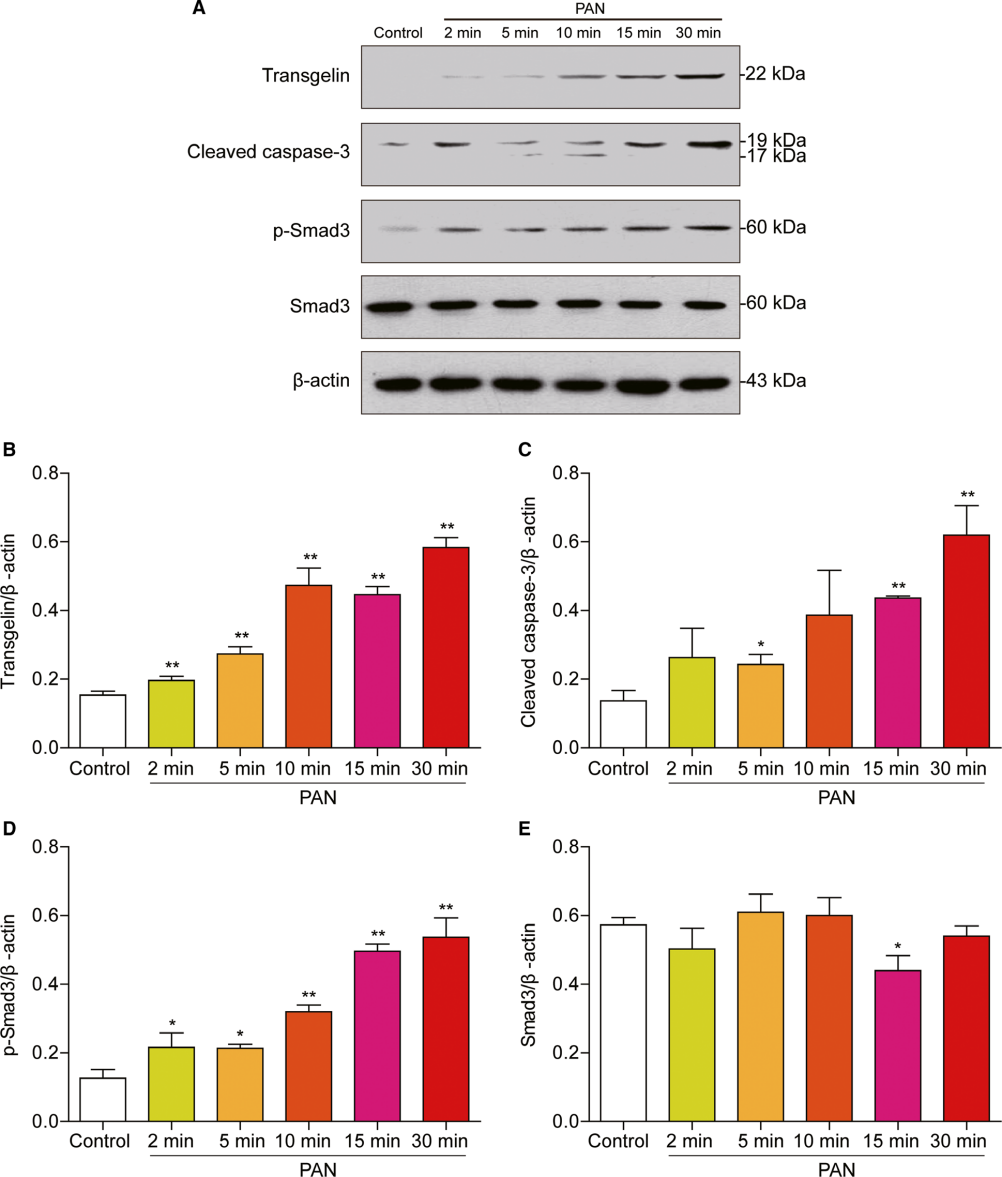
Expression of podocyte cytoskeletal proteins after PAN treatment. Podocytes were treated with PAN (50 μg·mL^−1^) for 0, 2, 5, 10, 15, and 30 min. (A) Representative immunoblots of transgelin, cleaved caspase 3, phosphorylated Smad3, and total Smad3 in total cell lysates. Induced expression of transgelin and phosphor‐Smad3 was observed after treatment with PAN. (B–E) Relative quantification of immunoblots with transgelin, cleaved caspase 3, phosphorylated Smad3, and total Smad3 antibodies (*n* = 3, means ± SEM). Data are representative of three independent experiments and were analyzed using one‐way ANOVA with Dunnett's multiple comparison test. *P < 0.05 and **P < 0.01 vs. control.

To confirm the increased expression of transgelin in PAN‐induced nephrotoxicity, MPC5 cells were treated with PAN for 2, 5, 10, 15, and 30 min. Total cell lysates were collected for analysis. Immunoblot analysis showed that transgelin expression and Smad3 phosphorylation were induced at the 10‐min time point of PAN treatment, and continued for 30 min (Fig. 2A–E). Cleaved caspase 3 was clearly detected at 30 min after PAN treatment. Expression of the total Smad3 protein was not induced by PAN at any time point.

### SIS3 downregulated the expression of transgelin

**Fig. 3:**
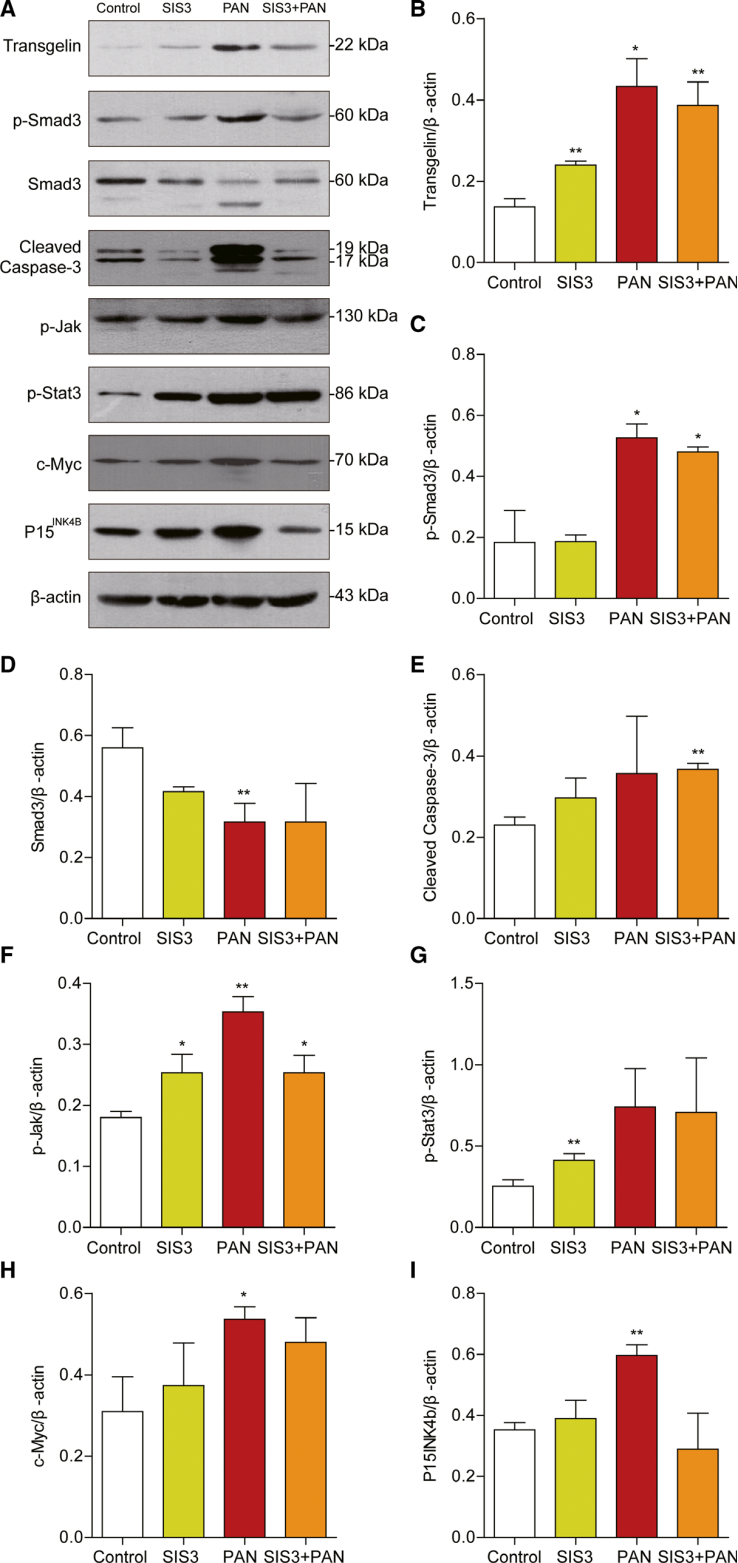
Expression of transgelin and TGF‐β signaling proteins upon inhibition of the Smad3 signaling pathway. After the differentiated MPC5 podocytes had grown to about 60% confluence, SIS3 (5 μm), a Smad3 inhibitor, was added to the culture medium 30 min before stimulation with PAN. Then, the podocytes were treated with PAN (50 μg·mL^−1^) for 12 h. (A) Representative immunoblots of transgelin, p15*^INK4B^*, phosphor‐smad3, phosphor‐JAK/stat3, cleaved caspase 3, and c‐myc in total cell lysates. Expression was examined using western blotting. (B–I) Relative quantification of immunoblots with transgelin, p‐smad3, total smad3, cleaved caspase 3, p‐JAK, p‐stat3, c‐myc, and p15*^INK4B^* antibodies (*n* = 3, means ± SEM). Data are representative of three independent experiments and were analyzed using one‐way ANOVA with Dunnett's multiple comparison test. *P < 0.05 and **P < 0.01 vs. control.

To further confirm whether Smad3 signaling affects PAN‐induced transgelin expression, the Smad3 inhibitor SIS3 was added to the MPC5 culture medium 30 min before PAN treatment. The expression of transgelin, p15*^INK4B^*, phosphor‐smad3, phosphor‐JAK/stat3, the apoptotic marker cleaved caspase 3, and c‐myc were induced significantly by PAN at 12 h (Fig. 3A–I). The effects of PAN induction were dramatically blocked by SIS3.

**Fig. 4:**
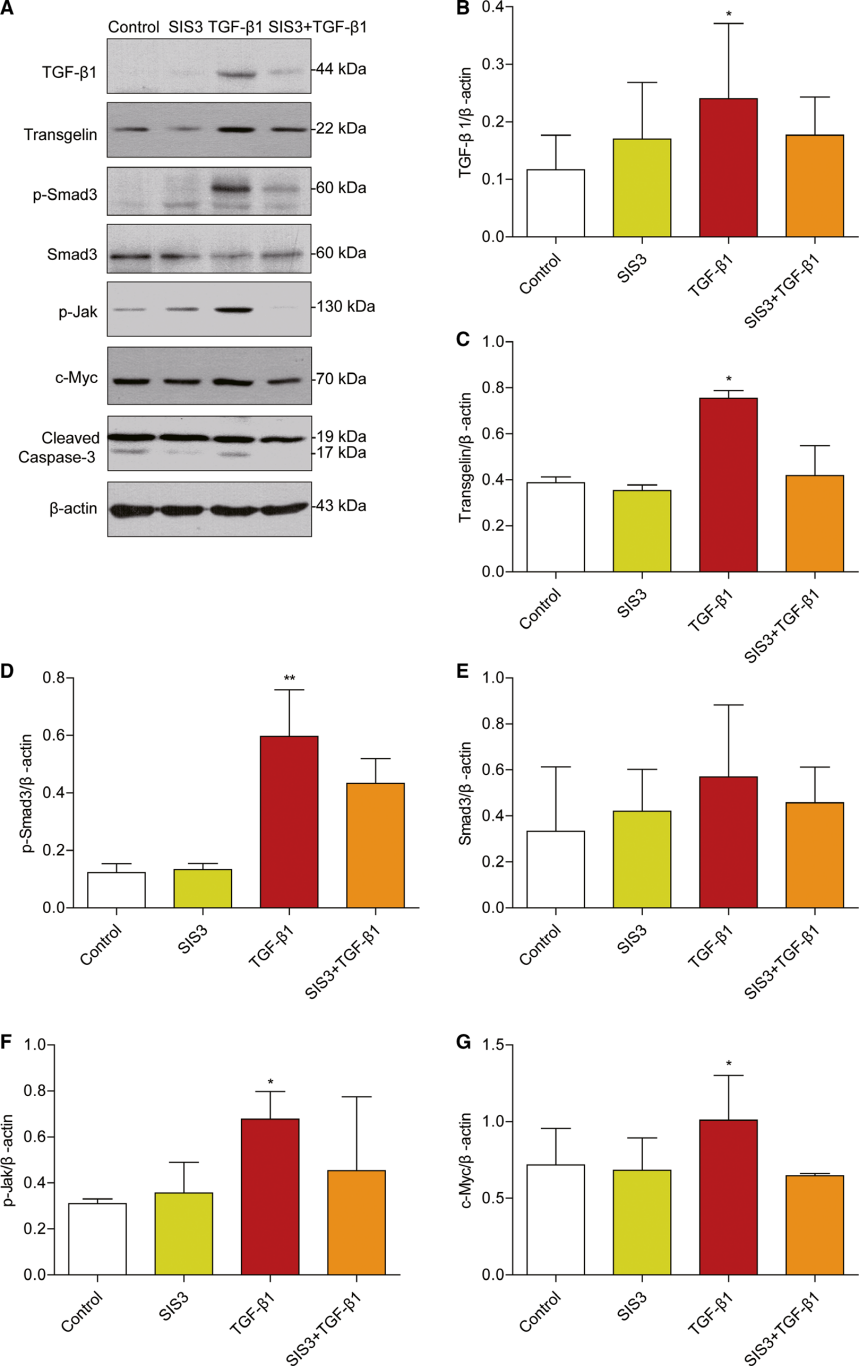
Expression of transgelin and TGF‐β signaling proteins after TGF‐β1 treatment. The 80% confluent differentiated podocytes were first cultured with 0.5% serum for 6 h and then treated with 5 μm SIS3 for 30 min and with 10 ng·mL^−1^ TGFβ1 for 24 h. (A) Representative immunoblots of transgelin, phosphor‐smad3, phosphor‐JAK/stat3, cleaved caspase 3, and c‐myc in total cell lysates. Expression was examined using western blotting. (B–G) Relative quantification of immunoblots with TGFβ1, transgelin, p‐smad3, total smad3, p‐JAK, and c‐myc antibodies (*n* = 3, means ± SEM). Data are representative of three independent experiments and were analyzed using one‐way ANOVA with Dunnett's multiple comparison test. *P < 0.05 and **P < 0.01 vs. control.

Since TGFβ1‐smad3 signaling pathway was activated during PAN‐induced MPC5 apoptosis, we wonder whether stimulating podocytes directly with TGFβ‐1 could also affect the expression of transgelin or phosphor‐JAK/stat3. For the group treated with the Smad3 inhibitor SIS3, 80% confluent differentiated podocytes were first cultured with 0.5% serum for 6 h and then treated with 5 μm SIS3 for 30 min and with 10 ng·mL^−1^ TGFβ1 for 24 h. Indeed, the expression of transgelin, phosphor‐smad3, phosphor‐JAK/stat3 was induced significantly by TGFβ1 (Fig. 4A–G). The effects of TGFβ1 induction were dramatically blocked by SIS3.

### SIS3 reversed the abnormal distribution of F‐actin during PAN‐induced podocyte injury

**Fig. 5:**
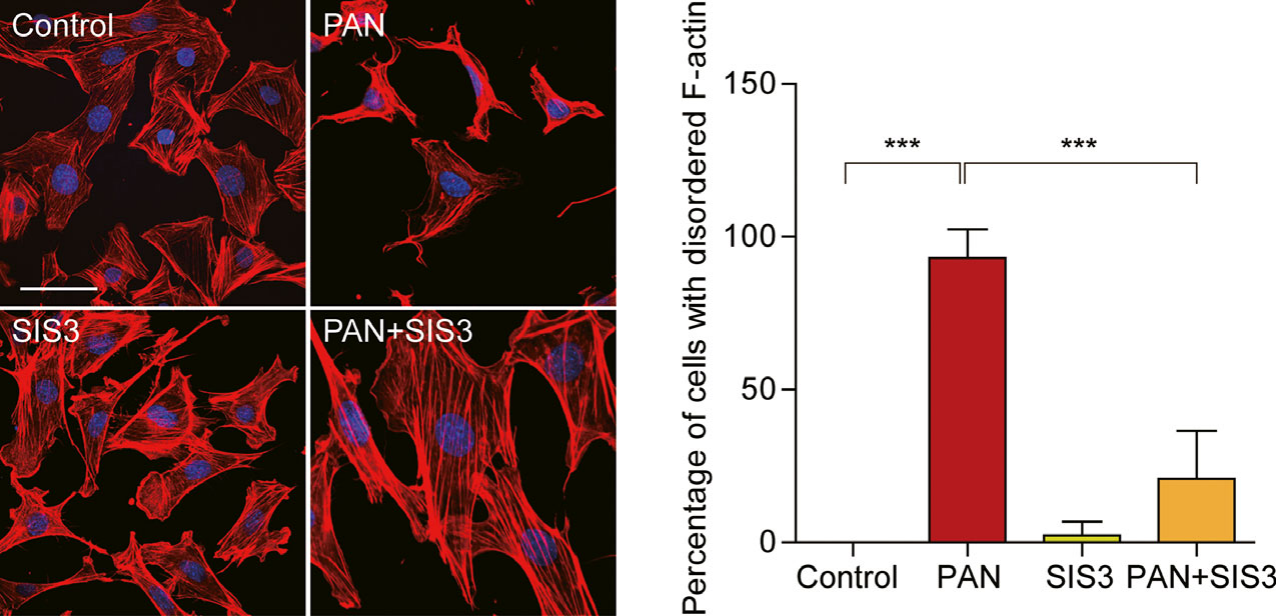
Distribution of F‐actin protein upon inhibition of the Smad3 signaling pathway. Control or SIS3 group: untreated control podocytes display well‐organized F‐actin. PAN group: Depolymerized and disordered F‐actin was observed in podocytes treated with PAN. SIS3 with PAN group: The Smad3 inhibitor SIS3 had no effect on F‐actin organization and appeared to block the disruptive effects of PAN. Scale bar = 50 μm. The percentage ratios of shrunken cell numbers to total cell numbers in the four groups are summarized in a histogram (*n* = 6, means ± SEM). ***P < 0.001.

Immunofluorescence staining showed that F‐actin was expressed primarily as fragmentary filaments in the cytoplasm in untreated podocytes (Fig. 5). After PAN treatment for 48 h, F‐actin staining was severely decreased and disordered. This change was significantly blocked by SIS3, which reversed the disorganization of the F‐actin cytoskeleton.

## Discussion

In the present study, we documented the protective effect of the Smad3 inhibitor SIS3 on PAN‐induced podocyte injury. PAN treatment induced transgelin expression and Smad3 phosphorylation and disordered the distribution of the podocyte cytoskeletal protein F‐actin. SIS3 restored F‐actin arrangement and the transgelin expression level. These results suggest that SIS3 alleviates podocyte injury in PAN‐induced nephrotoxicity by modulating transgelin expression and stabilizing F‐actin.

In a previous study, we identified the new podocyte cytoskeletal protein transgelin in a rat model of PAN‐induced kidney disease using high‐throughput microarray analysis [20]. The amino terminal domain of the transgelin protein sequence contains a CH structure, which also exists in the molecular structures of many actin‐binding proteins. This CH domain has been shown to aid localization of the actin cytoskeleton. The transgelin protein SM22α was shown to be involved in the regulation of actin cytoskeleton dynamics in mammalian smooth muscle [26].

In the present study, the expression of transgelin was enhanced during PAN‐induced podocyte injury. Increased transgelin expression has also been reported in other types of podocyte injury. In the case of atherosclerotic lesions, transgelin is involved in the regulation of phenotype changes (from proliferative to contractile) in vascular smooth muscle [27]. Transgelin was also induced in glomerulus epithelial cells in an FSGS model [23]. Recently, transgelin was reported to be highly expressed in podocytes during passive Heymann nephritis (experimental MN) [28]. In this study, increased transgelin expression was detected as early as 12 h after PAN treatment. In a unilateral ureteric obstruction model, transgelin expression was also induced early [26]. In patients with advanced pancreatic cancer, transgelin was upregulated and was an independent factor predictive of poor prognosis [29]. Altogether, these data suggest that transgelin can be used as a predictable marker of podocyte injury.

Puromycin aminonucleoside can activate the TGF‐β signaling pathway in podocytes [30]. TGF‐β is a key factor in the development of renal fibrosis, glomerulonephritis, and diabetic nephropathy. Peroxisome proliferator‐activated receptor agonists can protect against podocyte injury through blocking of the TGF‐β/Smad signaling pathway [31]. The Smad protein family plays a pivotal role in the transmission of TGF‐β intracellular signals [32]. The promoter region of the transgelin protein contains cis‐regulatory elements, including two serum response factor‐binding sites (the CarG box), three YY1‐binding sites, Sp1‐ and AP2‐binding sites, a TGF‐β control element (TCE), and a TATA box. These domains may be key components in the maintenance of tissue‐specific expression of the transgelin gene [33]. TCE mutation can completely abolish promoter activity. Activation of the transgelin promoter (−162 to 41 bp) by TGF‐β signaling depends on the phosphorylation of Smad proteins [30]. Induced by TGF‐β, Smad1, Smad3, and Smad4 interact and form heterotrimeric complexes, translocate to cell nuclei, and activate gene transcription by binding to the Smad binding site and MEDEA box in the transgelin promoter region. Smad3 plays the most important role in transgelin gene activation in this process. Transgelin, a TGF‐β‐inducible gene, has been reported to regulate actin cytoskeleton organization during the differentiation of human skeletal stem cells [34]. Wang *et al*. [35] observed the alleviation of diabetic glomerulosclerosis in Smad3‐knockout mice, but the persistence of proteinuria and FP effacement, indicating that the effect of Smad3 on the actin cytoskeleton is dependent on PAN stimulation.

Transgelin has been identified as a direct target of TGF‐β/Smad3 signaling in alveolar epithelial type II cells in lung fibrosis [36]. Chromatin immunoprecipitation assays indicated that Smad3 could bind to the transgelin promoter [27]. Such binding was also observed during myofibroblast differentiation [37], and Smad3 was found to be a major mediator of TGF‐β1‐induced transgelin promoter activation [28]. Interestingly, transgelin can, in turn, regulate Smad signaling. A transgelin expression intervention reduced TGF‐β1‐induced Smad signaling in lung epithelial cells [38]. Thus, TGF‐β and transgelin signaling may be regulated through a negative feedback mechanism.

In addition to active Smads, such as Smad2/3, inhibitory Smads regulate TGF‐β signaling. Smad6 can enhance Smad3 signaling in mesangial cells; Smad7 inhibits the Smad2/3‐mediated TGF‐β signaling pathway in podocytes and is induced during podocyte injury [39]. In addition, the TGF‐β family protein BMP7‐activated Smads 1, 5, and 8 showed the ability to restore podocyte injury caused by high glucose concentrations [40]. BMP7 was also reported to activate Smad5, but not Smad1, thereby promoting cell survival and podocyte differentiation, in a diabetic podocyte injury model [41]. In the present study, the expression of the TGF‐β downstream protein p15*^INK4B^* was induced upon PAN treatment. These data suggest that TGF‐β signaling is critically regulated and that many downstream pathways are involved in podocyte injury. In addition, in the PAN‐induced podocyte injury model, the Smad3 inhibitor obviously inhibited the Jak/STAT3 signaling pathway, indicating that the cytokine‑mediated inflammatory response may also be involved in the process. SIS3 significantly reversed PAN‐induced podocyte cytoskeleton disorder and transgelin expression, indicating that transgelin and Smad3 signaling are both important in PAN‐induced podocyte injury and are possible targets for future treatment of glomerulus albuminuria.

## Conclusion

During PAN‐induced podocyte injury, podocyte cytoskeleton damage, FP retraction, and pathological remodeling were observed. Additionally, transgelin expression was upregulated and mainly colocalized with F‐actin. Smad3 signaling was also activated in the process. SIS3, a Smad3 inhibitor, reversed the cytoskeletal disorder, the abnormal distribution of the microfilament skeleton protein F‐actin, and the upregulation of transgelin expression, suggesting that it has the potential to prevent podocyte damage.

## Conflict of interest

The authors declare no conflict of interest.

## Author contributions

LJ and JD conceived and designed research; LJ collected data and conducted research; LJ, YZ and AY analyzed and interpreted data; LJ wrote the initial paper; HC and JD revised the paper; LJ had primary responsibility for final content. All authors read and approved the final manuscript.

## Data Availability

The datasets generated and analyzed during the current study are available from the corresponding author on reasonable request.
